# Intestinal Barrier Dysfunction in the Absence of Systemic Inflammation Fails to Exacerbate Motor Dysfunction and Brain Pathology in a Mouse Model of Parkinson's Disease

**DOI:** 10.3389/fneur.2022.882628

**Published:** 2022-05-18

**Authors:** Aeja Jackson, Phillip A. Engen, Christopher B. Forsyth, Maliha Shaikh, Ankur Naqib, Sherry Wilber, Dulce M. Frausto, Shohreh Raeisi, Stefan J. Green, Brinda Desai Bradaric, Amanda L. Persons, Robin M. Voigt, Ali Keshavarzian

**Affiliations:** ^1^Rush Medical College, Rush Center for Integrated Microbiome and Chronobiology Research, Rush University Medical Center, Chicago, IL, United States; ^2^Department of Medicine, Rush University Medical Center, Chicago, IL, United States; ^3^Department of Anatomy and Cell Biology, Rush University Medical Center, Chicago, IL, United States; ^4^Genomics and Microbiome Core Facility, Rush University Medical Center, Chicago, IL, United States; ^5^Bachelor of Science in Health Sciences Program, College of Health Sciences, Rush University Medical Center, Chicago, IL, United States; ^6^Center for Compulsive Behavior and Addiction, Rush University Medical Center, Chicago, IL, United States; ^7^Department of Physician Assistant Studies, Rush University Medical Center, Chicago, IL, United States; ^8^Department of Physiology, Rush University Medical Center, Chicago, IL, United States

**Keywords:** Parkinson's disease, intestinal hyperpermeability, dextran sodium sulfate (DSS), microbiome, gut-brain axis

## Abstract

**Introduction:**

Parkinson's disease (PD) is the second most common neurodegenerative disease associated with aging. PD patients have systemic and neuroinflammation which is hypothesized to contribute to neurodegeneration. Recent studies highlight the importance of the gut-brain axis in PD pathogenesis and suggest that gut-derived inflammation can trigger and/or promote neuroinflammation and neurodegeneration in PD. However, it is not clear whether microbiota dysbiosis, intestinal barrier dysfunction, or intestinal inflammation (common features in PD patients) are primary drivers of disrupted gut-brain axis in PD that promote neuroinflammation and neurodegeneration.

**Objective:**

To determine the role of microbiota dysbiosis, intestinal barrier dysfunction, and colonic inflammation in neuroinflammation and neurodegeneration in a genetic rodent model of PD [α-synuclein overexpressing (ASO) mice].

**Methods:**

To distinguish the role of intestinal barrier dysfunction separate from inflammation, low dose (1%) dextran sodium sulfate (DSS) was administered in cycles for 52 days to ASO and control mice. The outcomes assessed included intestinal barrier integrity, intestinal inflammation, stool microbiome community, systemic inflammation, motor function, microglial activation, and dopaminergic neurons.

**Results:**

Low dose DSS treatment caused intestinal barrier dysfunction (sugar test, histological analysis), intestinal microbiota dysbiosis, mild intestinal inflammation (colon shortening, elevated MPO), but it did not increase systemic inflammation (serum cytokines). However, DSS did not exacerbate motor dysfunction, neuroinflammation (microglial activation), or dopaminergic neuron loss in ASO mice.

**Conclusion:**

Disruption of the intestinal barrier without overt intestinal inflammation is not associated with worsening of PD-like behavior and pathology in ASO mice.

## Introduction

Studies from our group and others support a role for the microbiome and intestinal tract (gut) in Parkinson's disease (PD) (1, 2). This model is known as the “gut-brain axis” (GBA) which is a bi-directional communication axis involving the intestinal microbiome, the intestinal barrier, intestinal inflammation, and the intestinal/systemic/brain immune systems (among other components) (3, 4). The gut-brain axis contributes to normal function and pathology of the central nervous system (4, 5). PD patients have an abnormal gut-brain axis (6–10).

PD patients have intestinal barrier dysfunction (6–10). Under normal conditions, the pro-inflammatory contents of the intestine are retained within the lumen of the intestine by the intestinal barrier which is comprised of both physical (mucus, tight junction proteins) and chemical (anti-microbial peptides) components. The barrier can become dysfunctional permitting the entrance of pathogenic bacteria and bacterial components including lipopolysaccharide (LPS) into the intestinal mucosa and the systemic circulation, prompting mucosal and systemic inflammation (1, 6, 11, 12), which may promote neuroinflammation, a key feature of PD.

In 2015, it was reported that patients with PD have intestinal microbiota dysbiosis (13, 14) and more than 20 studies since then have similarly demonstrated that the intestinal microbiome in PD patients is distinct from age matched subjects without PD (6–10). Although there is no unique PD microbiota signature, studies show that the dysbiosis in PD is characterized by an increased relative abundance of “putative” pro-inflammatory bacteria especially LPS-containing, Gram-negative bacteria and reductions in the relative abundance of putative anti-inflammatory bacteria [e.g., short chain fatty acid (SCFA)-producing bacteria] (1, 3, 15, 16). The pro-inflammatory microbiota can cause intestinal barrier dysfunction and disruption of the intestinal barrier can impact the microbiota leading to a positive feedback loop.

Intestinal barrier dysfunction and microbiota dysbiosis appear to be biologically meaningful. Studies demonstrate that the abundance of pro-inflammatory, LPS-containing, Gram-negative bacteria in PD subjects correlates with motor impairment in PD patients (1, 14, 17). Additionally, LPS is associated with more severe neuroinflammation in animal models of PD (8, 15, 18), and administration of LPS to mice is used as a model for neurodegeneration and PD (19–21). Taken together, studies suggest that the gut microbiota and microbiota-derived, pro-inflammatory molecules like LPS may contribute to PD pathogenesis.

One consequence of intestinal barrier dysfunction and microbiota dysbiosis is intestinal inflammation (1). Indeed, intestinal (e.g., stool calprotectin) and systemic (IL-1β, IL-6, and TNF-α) inflammation are reported in patients with PD and animal models of PD (8, 9, 22–26). Furthermore, inflammatory bowel disease (IBD), characterized by intestinal barrier dysfunction, pro-inflammatory changes in the intestinal microbiome, and chronic intestinal and systemic inflammation (27), is a risk factor for PD (27–29). This suggests that the inflammatory consequences of intestinal barrier dysfunction and intestinal microbiota dysbiosis are important in PD.

Intestinal microbiota dysbiosis, intestinal barrier dysfunction, and inflammation typically occur together in both animal models of PD and PD patients, therefore it is not clear which one these three elements is a primary driver of neuroinflammation and neurodegeneration in PD or whether all three “pro-inflammatory” factors are required. This study determined whether disruption of intestinal barrier/dysbiosis without significant intestinal inflammation was sufficient to worsen neuroinflammation and neurodegeneration in an animal model of PD.

## Methods And Materials

### Mice

Transgenic mice overexpressing human wild type α-synuclein under the Thy1 promoter were used for this study, known as ASO or “Line 61” mice (16, 30). Mice hemizygous for Thy1-α-synuclein overexpression were maintained on a mixed C57BL/6-DBA/2 background by breeding female BDF1 background, Thy1-α-synuclein animals hemizygous for the Thy1-α-synuclein transgene on the X-chromosome with wild-type male BDF1 (Charles River, Wilmington, USA) to generate the male ASO and control littermates (without the transgene). Breeding pairs were replenished every 6 months with transgenic females and newly generated BDF1 males. The genotype of ASO and control mice was verified with PCR (16). The transgene is inserted in the X chromosome, which undergoes random chromosomal silencing, so only male mice are used experimentally (30).

Mice were maintained on a 12 h light/dark cycle with free access to water and food and were singly housed. All animal husbandry and experiments were approved by the Rush University Institutional Animal Care and Use Committee (IACUC).

### Dextran Sodium Sulfate Administration

**Figure 1:**
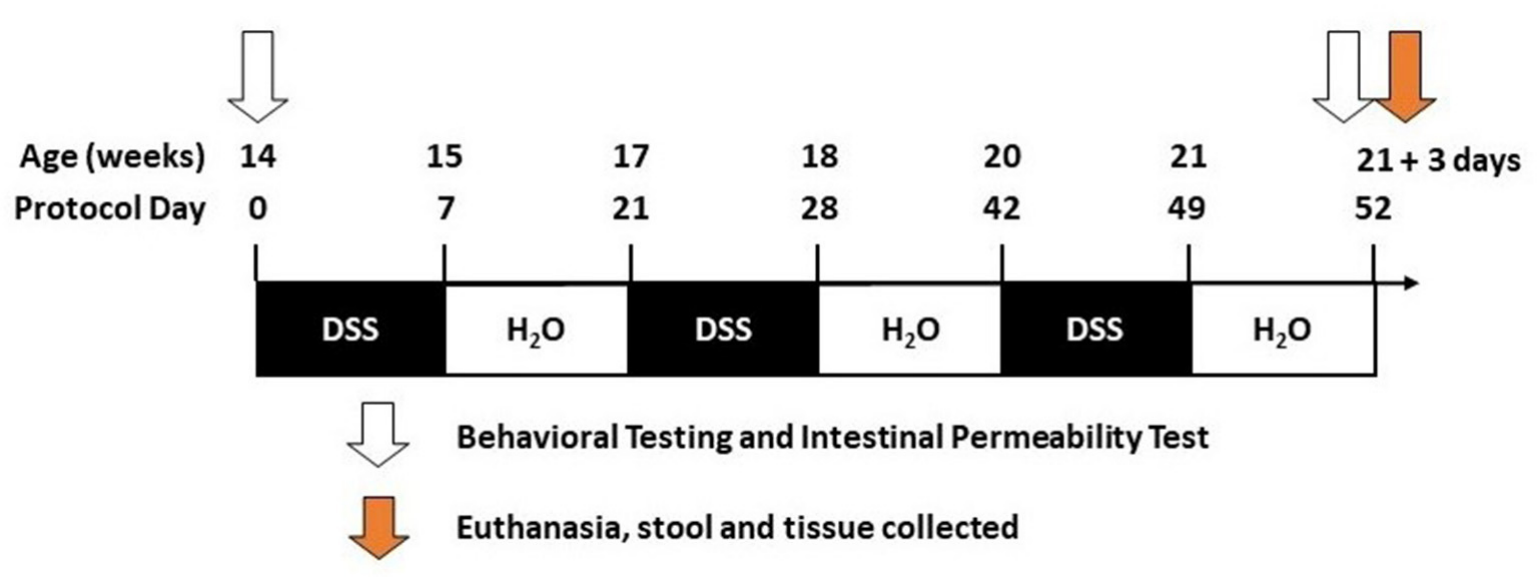
Timeline. The diagram illustrates the dextran sodium sulfate (DSS) treatment over given over three cycles during the 52 days of the study (see Methods).

DSS has a direct toxic effect on intestinal epithelial cells leading to disruption of the intestinal barrier (31–33). DSS (molecular weight 36,000–50,000, MP Biomedicals, Santa Ana, CA) was given to mice in filtered drinking water and was replenished every other day. DSS was administered beginning when mice were 14 weeks of age and was given over three cycles. A DSS cycle is defined by 7 days on DSS followed by a 14-day recovery period with no DSS (21-day cycle; Figure 1). Vehicle-treated mice (i.e., H_2_O) were given only drinking water (i.e., without DSS).

### Tissue Collection and Processing

Tissue was collected 3 days after the last DSS cycle (Figure 1). Necropsy was performed under anesthesia as approved by Rush IACUC#18–052. Mice were deeply anesthetized (90 mg/kg ketamine, 10 mg/kg xylazine in a 0.9% saline diluent). Blood was collected by cardiac puncture and stored on ice until serum isolation/collection. After blood collection, mice were perfused with cold PBS. The abdomen was clamped using hemostatic forceps to perfuse the upper body. The brain was collected and immersion-fixed in 4% paraformaldehyde, intestinal tissue was measured for length (end of the cecum to the anus), and colon samples were collected and stored either in optimal cutting temperature (OCT) media (4583, Tissue-Tek), 4% paraformaldehyde, or flash frozen in liquid nitrogen.

### Intestinal Barrier Integrity

The oral sugar test was used to assess region-specific differences in intestinal barrier integrity (34–36). We have previously published that sucralose, and especially the Sucralose/Lactulose ratio is primarily a marker of the colonic permeability, including in PD patients (35). Lactulose and mannitol are markers of small intestinal permeability with an elevated Lactulose/Mannitol ratio indicating the small intestinal barrier hyperpermeability (36).

*In vivo* intestinal barrier integrity was assessed at baseline (14 weeks of age) and the end of the last DSS cycle (22 weeks of age) as previously described (8, 35). Briefly, mice were fasted for 8 h prior to the test. A 200 ul solution containing lactulose (3.2 mg), sucrose (0.45 mg), sucralose (0.45 mg), and mannitol (0.9 mg) was administered *via* gavage, after which 2 ml of 0.9% saline was administered subcutaneously to promote urine output. Mice were placed in metabolic cages and urine was collected for 5 h and the total volume recorded. Intestinal permeability was calculated by measuring urinary sugar concentration with gas chromatography which is expressed as percent excretion of the oral dose of sugar (8, 36).

### Immunofluorescent Staining

#### Gastrointestinal Tissue

The integrity of the intestinal barrier is maintained by a series of inter-locking proteins between intestinal epithelial cells known as the Apical Junctional Complex (AJC) (37, 38). This AJC is composed of tight (zonula adherens 1, ZO-1) and adherens (E-cadherin) junctions which were examined in this study (38, 39). OCT-embedded intestinal tissue (ZO-1) was cut into 5 μm sections, and then fixed using acetone at −20° for 20 min. Paraffin-embedded intestinal tissue (E-Cadherin) was cut into 5 μm sections, which were de-paraffinized and rehydrated using serial ethanol dilutions (100, 95, and 70%) (40). Heat-induced antigen retrieval was completed by submerging tissue in an EDTA buffer for 4 min using a pressure cooker. Slides were blocked with 10% donkey serum (Jackson ImmunoResearch, 017-000-12) overnight, followed by overnight incubation with antibody (ZO-1: 1:500 Invitrogen #61-7300; E-Cadherin: 1:500 Cell Signaling #14472). Secondary antibody diluted at 1:250 (Alexa Fluor 555, #4409) was applied for 45 min, followed by washing. Sections were then DAPI-stained and mounted using Fluoromount Aqueous Mounting Medium (Sigma-Aldrich, #F4680). Immunofluorescence images were acquired using a Zeiss Axio Observer 7 at 20x magnification, two images per sample (40).

#### Brain Tissue

Ionized calcium binding adaptor molecule-1 (Iba-1) is a microglia/macrophage-specific calcium-binding protein that is a widely validated marker for microglia identification and microglial morphology characterization (41). Tyrosine hydroxylase (TH) is the rate-limiting enzyme of catecholamine biosynthesis and a robust marker of dopaminergic neurons (40, 42). Loss of TH staining in the striatum is a hallmark for loss of dopaminergic terminals that is characteristic of neurodegeneration in PD (43). Brain tissue was cut at 30 μm thickness using a cryostat (CM3050, Leica) and was stored in cryoprotectant until analysis (40). In brief, sections were washed with dilution media for 60 min. An antigen retrieval step was performed using a citric acid buffer solution (6.0 pH) for 20 min. Then, an endogenous sodium peroxidase block was performed using a sodium periodate solution for 20 min. Following peroxidase blocking, sections were washed multiple times in dilution media and incubated in serum blocking solution for an hour (2% BSA and 3% serum targeting host of the secondary antibody). Sections were incubated in primary antibody (Iba-1: 1:1000, Wako 019–19741; TH: 1:10,000, Immunostar 22941) overnight at room temperature. The next day, sections were washed and processed with biotinylated secondary antibodies (1:200, Vector Laboratories BA1000, BA2000). Immunoperoxidase sections were treated with a standard ABC HRP Biotin/Avidin Complex Kit (Vector Laboratories). Incubation was performed before developing a color reaction in the presence of DAB chromogen and hydrogen peroxide. Once completed, immunoperoxidase stained sections were mounted on glass slides, cover-slipped using Cytoseal TM 60 mounting medium (8310-16) and analyzed.

### Western Blot Analysis

#### Isolation of Nuclear and Cytoplasmic Extracts and Analysis

The cytoplasmic and membrane extraction was prepared using an NE-PER Nuclear Cytoplasmic Extraction Reagent kit (Pierce, Rockford, IL, USA) as previously described (36). Briefly, tissue was washed twice with cold PBS and centrifuged at 500 × *g* for 5 min. The pellet was suspended in 200 μl of cytoplasmic extraction reagent I by vortexing. The suspension was incubated on ice for 10 min followed by the addition of 11 μl of cytoplasmic extraction reagent II, vortexed for 5 s, incubated on ice for 1 min and centrifuged for 5 min at 16,000 × *g*. The supernatant fraction (cytoplasmic extract) was transferred to a pre-chilled tube. The insoluble pellet fraction, which contains crude nuclei, was resuspended in 25 μl of nuclear extraction reagent by vortexing during 15 s and incubated on ice for 10 min, then centrifuged for 10 min at 16,000 × *g*. The remaining insoluble pellet, containing membrane fragments, was suspended in 100 μl of tris-triton buffer. Samples were incubated on ice for 20 min and then centrifuged (16,000 × *g*, 10 min). The supernatant was collected and stored at −80°C.

#### Western Blot

Equal amounts of the protein concentrations were quantified and normalized to the β-actin band. Homogenized colon samples (30 μg) were boiled at 95°C for 5 min with 2x Laemmli sample buffer (Bio-Rad Laboratories, Hercules, CA). Samples were electrophoresed on 7.5% tris-HCl gels and transferred to a nitrocellulose membrane (GE Healthcare Limited, Buckinghamshire, UK). Non-specific binding sites were blocked for 1 h at room temperature {E-cadherin and ZO-1: 5% bovine serum albumin (BSA); β-actin: 2.5% BSA and 2.5% non-fat dry milk [all in tris-buffered saline / Tween-20 (TBS-T)]}. Membranes were incubated overnight at 4°C with primary antibody [E-cadherin: 1:1,000, Cell signaling 14472; ZO-1: 1:1,000, Invitrogen 61-7300; β-actin: 1:5,000, Sigma A2066 (all in TBS-T)]. Membranes were incubated in HRP-conjugated anti-rabbit secondary antibody (1:2,000) for 1 h at room temperature. Chemiluminescent substrate (ECL, GE Healthcare) was applied to the membrane for protein visualization using autoradiography film (HyBlot CL, Denville Scientific, Metuchen, NJ). Films and were scanned and optical density determined using ImageJ software (NIH, Bethesda, MD) (36, 44).

### Intestinal Inflammation

#### Myeloperoxidase (MPO)

MPO is a reliable and well-established marker of intestinal inflammation (45–47). Colon tissue was homogenized and MPO was quantified using the MPO enzyme-linked immunosorbent assay (ELISA) kit (Hycult Biotechnology, Uden, The Netherlands) according to the manufacturer's instructions (47). Briefly, 10 mg of colon tissue was homogenized in 200 μl lysis buffer. Then, sample aliquots were applied onto microtiter well-precoated with capture antibody. After washing, biotinylated tracer antibody was added to each well. After incubation, the color development with tetramethylbenzidine was performed and the color reaction was stopped by the addition of oxalic acid. Absorbance at 450 nm was measured with a spectrophotometer. MPO concentration of each sample was calculated from a standard curve (serial dilution).

#### Calprotectin

Calprotectin is produced by neutrophils in the intestine and is a reliable and well-accepted method to assess intestinal inflammation (48). PD patients also have increased levels of calprotectin (9, 25, 49). Cecal content of the mice was collected during tissue collection was stored at −80°C until used for this assay. Calprotectin ELISA was performed using S100A8/S100A9 Elisa kit (ref K6936) from Immunodiagnostik (Immunodiagnostik, Bensheim, Germany) following the manufacturer's protocol. The concentration of calprotectin was calculated from measured OD 450 nm values by the Gene5 program (Biotek, Winooski, VT) (50).

#### Hematoxylin and Eosin Histology

Formalin-fixed colon was stained with hematoxylin & eosin (H&E). Blinded assessment of samples was conducted by a gastrointestinal pathologist (SS). Histological analyses, including inflammatory cell infiltrate, epithelial changes, and the mucosal architecture, were scored according to an established criterion (51). Mild colonic inflammation is operationally defined in this study as an increase in MPO levels and decrease in colon length, without elevated fecal calprotectin values. We chose to use elevated stool calprotectin as part of our definition of severe intestinal inflammation because, according to the American College of Gastroenterology, fecal calprotectin levels are a sensitive and specific marker of intestinal inflammation. Indeed, evaluation of stool calprotectin level has become routine for many clinicians who are managing patients with intestinal inflammatory diseases, such as ulcerative colitis (52). Relevant to our study, stool calprotectin is routinely used to define intestinal inflammation in patients with inflammatory bowel disease (53–55) and in patients with Parkinson's disease (49, 56, 57).

### Microbial Translocation and Systemic Inflammation

#### LPS-Binding Protein (LBP)

LBP is a type 1 acute-phase protein that binds to LPS to facilitate an immune response that our group and others have shown is altered in PD patients with intestinal permeability (8, 58, 59). Serum collected at the time of cardiac puncture was used to measure systemic LBP levels using an LBP ELISA kit (HK205; Hycult Biotech) as previously described (8).

#### Cytokines

Serum cytokine levels were assessed with Meso Scale 10-plex V-PLEX Proinflammatory Panel 1 Mouse Kit (Cat. # K15048D, Meso Scale Diagnostics, Rockville, MD) as previously described (60).

### Motor Function

Motor performance and coordination were assessed at 14 and 22 weeks of age including adhesive removal, beam traversal, and hindlimb clasping reflex.

#### Adhesive Removal

This test evaluates somatosensory and motor function. A one-quarter inch round adhesive (Avery, Glendale, CA) was placed on the nasal bridge between the nostrils and the forehead of the mouse, and the time to make contact and remove the adhesive was recorded. All testing was performed in the home cage. If the mouse did not remove the adhesive within 60's, the trial was ended. Time to make contact/remove the adhesive was recorded over three trials (16).

#### Beam Transversal

This test assesses motor coordination and balance. A 1 m plexiglass beam (Stark's Plastics, Forest Park, OH) was used. The beam was constructed of four segments of 0.25 m in length with each segment having a progressively thinner width: 3.5, 2.5, 1.5, and 0.5 cm. The widest segment acted as the loading platform for the animals and the narrowest end was placed into the home cage. Mice had 2 days of training prior to testing. On the 1st day of training, mice received one trial with the home cage positioned close to the loading platform and the mice were guided forward along the narrowing beam. Mice received two more trials with limited or no assistance to encourage forward movement on the beam. On the 2nd day of training, mice had three trials to transverse the beam and generally did not require assistance in forward movement. On the 3rd day, mice were tested over three trials for time to transverse from the loading platform to the home cage. Timing began when mice placed their forelimbs onto the 2.5 cm segment and ended when one forelimb reached the home cage. Maximum test time (cut-off time) was 60 s, and the mice were videotaped. Videos were viewed in slow motion to count errors made by each mouse. An error was counted when, during forward movement, at least 50% of a limb (forelimb or hindlimb) slipped off the beam. Slips were not counted if the mouse was not making forward movement or when the mouse's head was oriented to the left or right of the beam. Percentage of misstep errors were calculated for control and ASO mice across all three trials and averaged (16).

#### Hindlimb Clasping Reflex

This reflex indicates uncoordinated movement and precedes the symptomatic onset of hindlimb paralysis. Mice were gently lifted upward by the mid-section of the tail and observed over ~5–10 s (16, 61). Mice were assigned a score of 0–3 based on the extent to which the hindlimbs clasped inward. A score of 0, indicating no clasping, was given to mice that freely moved both their limbs and extended them outward. A score of 1 was assigned to mice which clasped one hindlimb inward for the duration of the restraint or if both legs exhibited partial inward clasping. A score of 2 was given if both legs clasped inward for most of the observation, but still exhibited some flexibility. A score of 3 was assigned if mice displayed complete paralysis of hindlimbs that immediately clasped inward and exhibited no signs of flexibility.

### Stool Sample Collection and Microbiota Analyses

Mice stool pellets were collected over a 24 h period before tissue collection and stored at −80°C until analysis. Total genomic DNA was extracted from the mice feces using the FastDNA SPIN Kit from the manufacturer's protocol (FastDNA Spin Kit for Soil, MP Biomedicals, Solon, OH), and verified with fluorometric quantitation (Qubit 3.0, Life Technologies, Grand Island, NY, USA). To reduce batch effects, all samples were extracted using the same DNA extraction kit at the same time, and library preparation for all samples was conducted in 96-well plates simultaneously. Primers 515F/806R (515F: GTGTGYCAGCMGCCGCGGTAA; 806R: CCGGACTACNVGGGTWTCTAAT) modified from the Earth Microbiome Project primers, and targeting the V4 variable region of microbial 16S ribosomal RNA (rRNA) genes, were used for PCR, and prepared for high-throughput amplicon sequencing using a two-stage PCR method, as previously described (62). Sequencing was performed using an Illumina MiniSeq, with a V2 kit and paired-end 150 base reads at the Genomics and Microbiome Core Facility (GMCF) at Rush University Medical Center.

#### 16S rRNA V4 Sequencing Analysis

Raw sequences were merged using the software package PEAR (Paired-End read merger) algorithm (v0.9.11) (Dalhousie University, Halifax, Nova Scotia, Canada) (63). Merged sequences shorter than 240 bases were removed. Merged sequences were then processed (including denoising) using the DADA2 algorithm within the QIIME2 (v 2020.8.0) workflow (64, 65). The amplicon sequence variants (ASVs) generated were used for all downstream analyses. Taxonomy was assigned to each ASV using the na Bayes classifier employing the SILVA 138 99% OTUs reference database (66, 67). A total of 1,156,631 sequencing clusters were generated, with an average of 20,654 clusters per sample (median = 27,444; min = 0; max = 41,767). One reagent contaminant ASV (*Pseudomonas*) was identified and removed using decontam package based on the prevalence of the ASV in the reagent negative blank controls (*n* = 5), using default parameters (68). Unassigned, eukaryote, chloroplast, and mitochondrial ASVs were removed from datasets prior to statistical analyses (69). Raw sequence data were deposited in the NCBI Sequence Read Archive under BioProject PRJNA781983.

### Statistical Analysis

#### Experimental and Behavioral Statistical Analyses

These data are reported as mean + standard error of the mean (SEM), unless otherwise stated. Differences among means were analyzed using GraphPad Prism (v9.3.1) software (GraphPad Software, La Jolla, CA). We removed outlier points by eliminating any points that were two standard deviations above and below the mean of each respective group. Two-way analysis of variance (ANOVA) was performed to evaluate the significant differences with genotype (control vs. ASO) or treatment (vehicle vs. DSS). Multiple group comparisons were performed using Tukey's *post-hoc* comparison. Pearson correlation analysis was performed to evaluate associations between intestinal permeability and brain-related outcomes. Significance was considered at the value *p* < 0.05 (16, 40).

#### Microbiota Statistical Analysis

Analyses of alpha- and beta-diversity were used to compare fecal microbial community structure. All analyses were performed on feature (ASV) counts. Alpha-diversity metrics (i.e., Shannon index, Simpson's index, Observed features, and Pielou's Evenness) were calculated on rarefied datasets (19,000 sequences/sample). Differences in alpha diversity were assessed for significance using the Mann-Whitney *U*-test (MWU) with Benjamini–Hochberg false-discovery rate (FDR) correction for multiple comparisons (*q* < 0.05). Analyses were performed using the software package GraphPad Prism (v9.3, GraphPad Software LLC San Diego California). Permutation Multivariate Analysis of Variance (PERMANOVA) with 9,999 permutations was used to assess global differences in microbial community structure between treatments (70). Adjustment for multiple testing was conducted using the Benjamini–Hochberg FDR correction. Visualization of data was performed using principal coordinates analysis (PCoA) based on a Bray–Curtis dissimilarity distance matrix within the software package QIIME2 (65). Differential abundance analyses of individual taxa between groups were performed using the software package DESeq2, generating an FDR *q*-value (71, 72). DESeq2 has been shown to be appropriate for differential abundance comparisons in studies with small sample size groups (<20) or unbalanced design (73). Individual taxa percent mean relative abundances (>1%) and standard deviations (SD) calculated and depicted as stacked histograms. To identify taxa that most strongly explained between group differences, a Linear discriminant analysis Effect Size (LEfSe) analysis was performed (74). LEfSe uses the non-parametric factorial Kruskal-Wallis sum-rank test to detect individual taxa that differ between treatments and animal genotype. Taxa that are significant by Kruskal-Wallis are subsequently investigated using a set of pairwise tests among subclasses using the (unpaired) Wilcoxon rank-sum test. As a last step, LEfSe uses Linear Discriminant Analysis to estimate the effect size of each differentially abundant taxa. Differentially abundant taxa that were statistically significant using an alpha of (0.05) and exceeded an LDA log score of at least (±2) were graphically represented.

## Results

### Effects of DSS on Intestinal Barrier Function

A dose response was conducted to identify an optimal dose of DSS that would be used for all subsequent experiments. Mice were given a range of DSS from 0.5 to 2% (given daily over three cycles) to determine the lowest dose that caused intestinal barrier dysfunction [sucralose/lactulose ratio (S/L)] without overt intestinal inflammation. Results demonstrated that the 0.5% dose of DSS did not induce intestinal barrier dysfunction and 2% induced overt intestinal inflammation whereas 1% DSS increased the S/L ratio without an increase in intestinal inflammation (i.e., calprotectin) data not shown. Therefore, 1% DSS was used for all experiments in this study.

**Figure 2:**
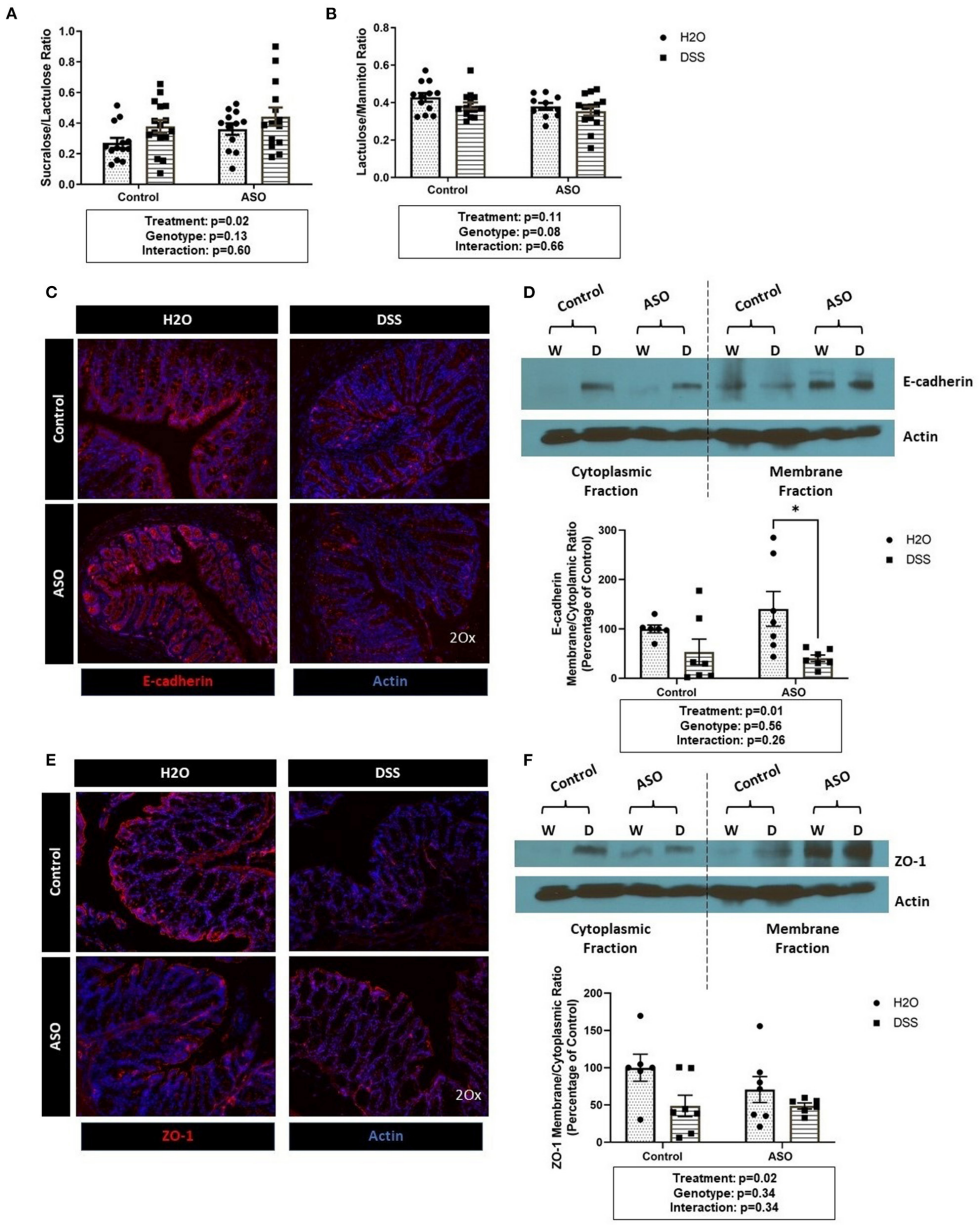
Impact of DSS on the intestinal barrier. **(A)** Mice treated with DSS exhibited intestinal barrier dysfunction, specifically in the colon, as assessed the by the sucralose/lactulose ratio. **(B)** Small intestinal permeability was similar in all groups regardless of genotype and treatment, based on the lactulose/mannitol ratio. **(C)** Immunofluorescent staining of E-cadherin (red) in colonic tissue (DAPI, blue) showing reduced staining in DSS treated tissue. **(D)** The membrane/cytoplasm ratio of E-cadherin showed that DSS-treated mice have less E-cadherin in the cytosol compared to H_2_O-treated mice. **(E)** Immunofluorescent staining of ZO-1 (red) in colonic tissue (DAPI, blue) shows reduced staining in DSS treated tissue. **(F)** There was a significant reduction of ZO-1 in the membrane/cytoplasm ratio in mice treated with DSS. Outliers were omitted prior to analysis (>2 standard deviations from the mean). Two-way ANOVA was conducted and values for different factors are indicated in the graphs followed by *post-hoc* Tukey which is indicated on each graph when appropriate *p < 0.05. ASO, α-synuclein overexpressing; W/H_2_O, water; DSS, dextran sodium sulfate.

DSS disrupted intestinal barrier integrity in the colon (i.e., large intestine) but not in the small intestine (Figures 2A,B). Specifically, DSS administration significantly increased the S/L ratio (Figure 2A: two-way ANOVA: genotype *p* = 0.13, treatment *p* = 0.02, interaction *p* = 0.60) without affecting urinary mannitol (data not shown), lactulose (data not shown), nor the lactulose/mannitol ratio (Figure 2B: two-way ANOVA: genotype *p* = 0.08, treatment *p* = 0.11, interaction *p* = 0.66).

Results from the sugar test indicated that DSS-induced intestinal barrier dysfunction primarily occurred in the colon, therefore the AJC proteins E-cadherin and ZO-1 were assessed in colon tissue. DSS administration reduced E-cadherin staining (Figure 2C) and caused a significant shift from E-cadherin from the membrane to the cytosolic fraction as indicated by the decrease in the membrane/cytoplasmic ratio which was observed in both control and ASO mice (Figure 2D: two-way ANOVA: genotype *p* = 0.56, treatment *p* = 0.01, interaction *p* = 0.26). Similarly, ZO-1 staining was reduced by DSS treatment (Figure 2E). The membrane/cytoplasmic ratio of ZO-1 was significantly reduced by DSS in both control and ASO mice (Figure 2F: two-way ANOVA: genotype *p* = 0.34, treatment *p* = 0.02, interaction *p* = 0.34).

Taken together the sugar test and the AJC protein data support that DSS induced intestinal barrier dysfunction in the colon (treatment effect), but these effects could not be distinguished based on genotype (i.e., control and ASO mice respond similarly to DSS).

### Effects of DSS on Intestinal Inflammation

**Figure 3:**
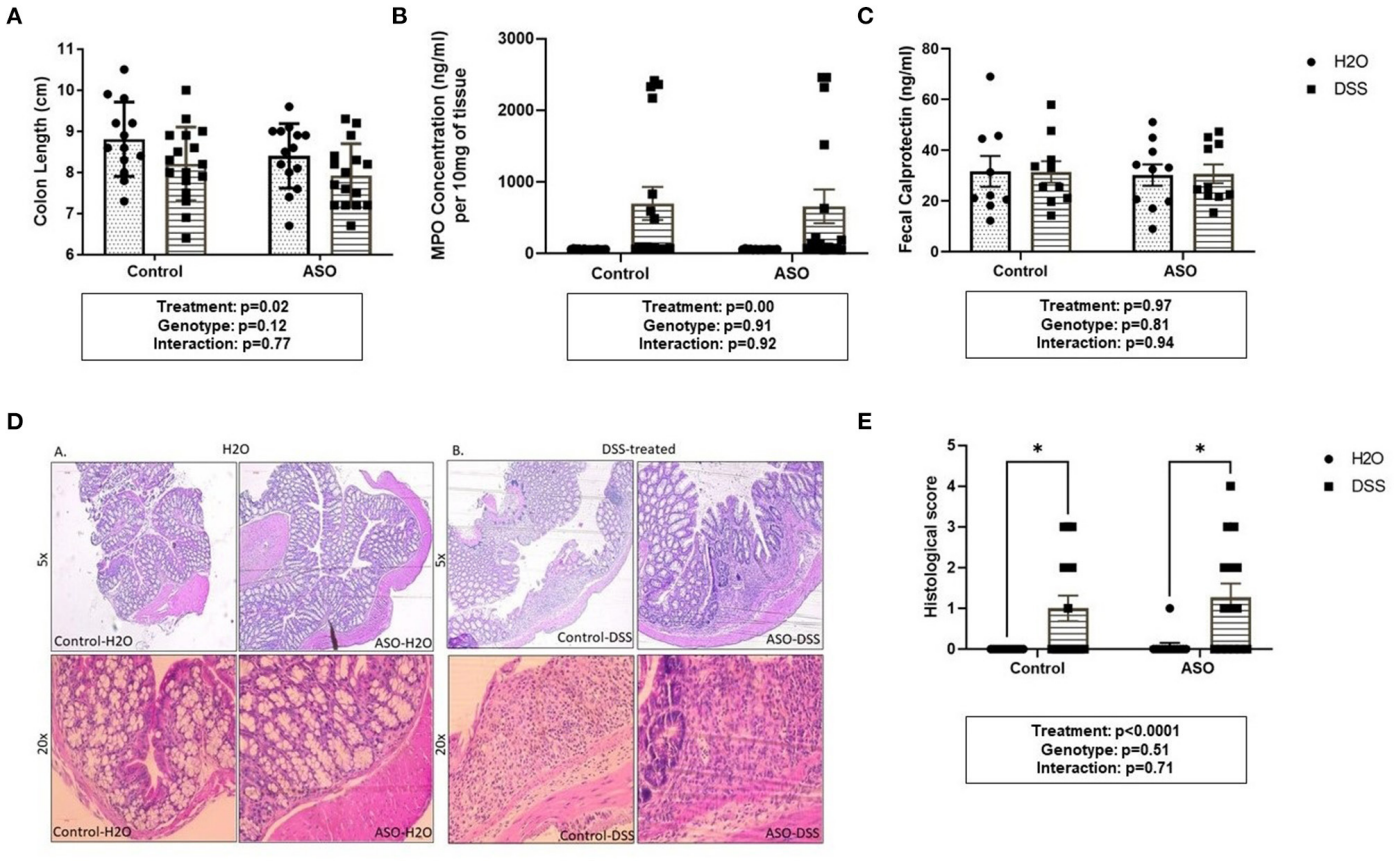
Measures of colon inflammation in DSS-treated control and ASO mice. We used three measures of colonic inflammation: colonic shortening, colonic MPO, and fecal calprotectin (see Methods). **(A)** DSS-treated mice had significantly shorter colons than mice on water, regardless of genotype. **(B)** Mice treated with DSS also had significantly higher colonic MPO levels compared to mice on water. **(C)** Fecal calprotectin levels were similar for WT and ASO mice, regardless of treatment. **(D)** H&E staining showed diffuse inflammatory infiltration in the mucosa and submucosa in DSS-treated mice, regardless of genotype. **(E)** DSS treatment was associated with increased histological score compared to H_2_O-treated mice, regardless of genotype. Outliers were omitted prior to analysis (>2 standard deviations from the mean). Two-way ANOVA was conducted and values for different factors are indicated in the graphs followed by *post-hoc* Tukey which is indicated on each graph when appropriate *p < 0.05. ASO, α-synuclein overexpressing; H_2_O, water; DSS, dextran sodium sulfate; MPO, Myeloperoxidase.

DSS caused mild colonic inflammation in both control and ASO mice (Figure 3). Specifically, DSS-treated control and ASO mice had shorter colon than vehicle treated mice which is consistent with intestinal inflammation (Figure 3A: two-way ANOVA: genotype *p* = 0.12, treatment *p* = 0.02, interaction *p* = 0.77). There was a concurrent increase in tissue MPO, marker of tissue inflammation in a subset of both control and ASO mice (Figure 3B: two-way ANOVA: genotype *p* = 0.91, treatment *p* < 0.00, interaction *p* = 0.92). The increase in MPO was significant but was driven by a few mice, with most mice not demonstrating an increase in MPO. Fecal calprotectin was not significantly increased by DSS (Figure 3C: two-way ANOVA: genotype *p* = 0.81, treatment *p* = 0.97, interaction *p* = 0.94). To support the tissue inflammation data, colonic tissue was stained with H&E and scored for intestinal inflammation by a pathologist. H&E staining showed diffuse inflammatory infiltration in the mucosa and submucosa in DSS-treated mice, regardless of genotype (Figures 3D,E). These data collectively showed that DSS administration induced colon shortening length and increase in MPO in a subset of mice but had no effect on fecal calprotectin which can be interpreted as mild colonic inflammation. Additionally, DSS had similar inflammatory effects on control and ASO mice.

### Effects of DSS on the Intestinal Microbiota

**Figure 4:**
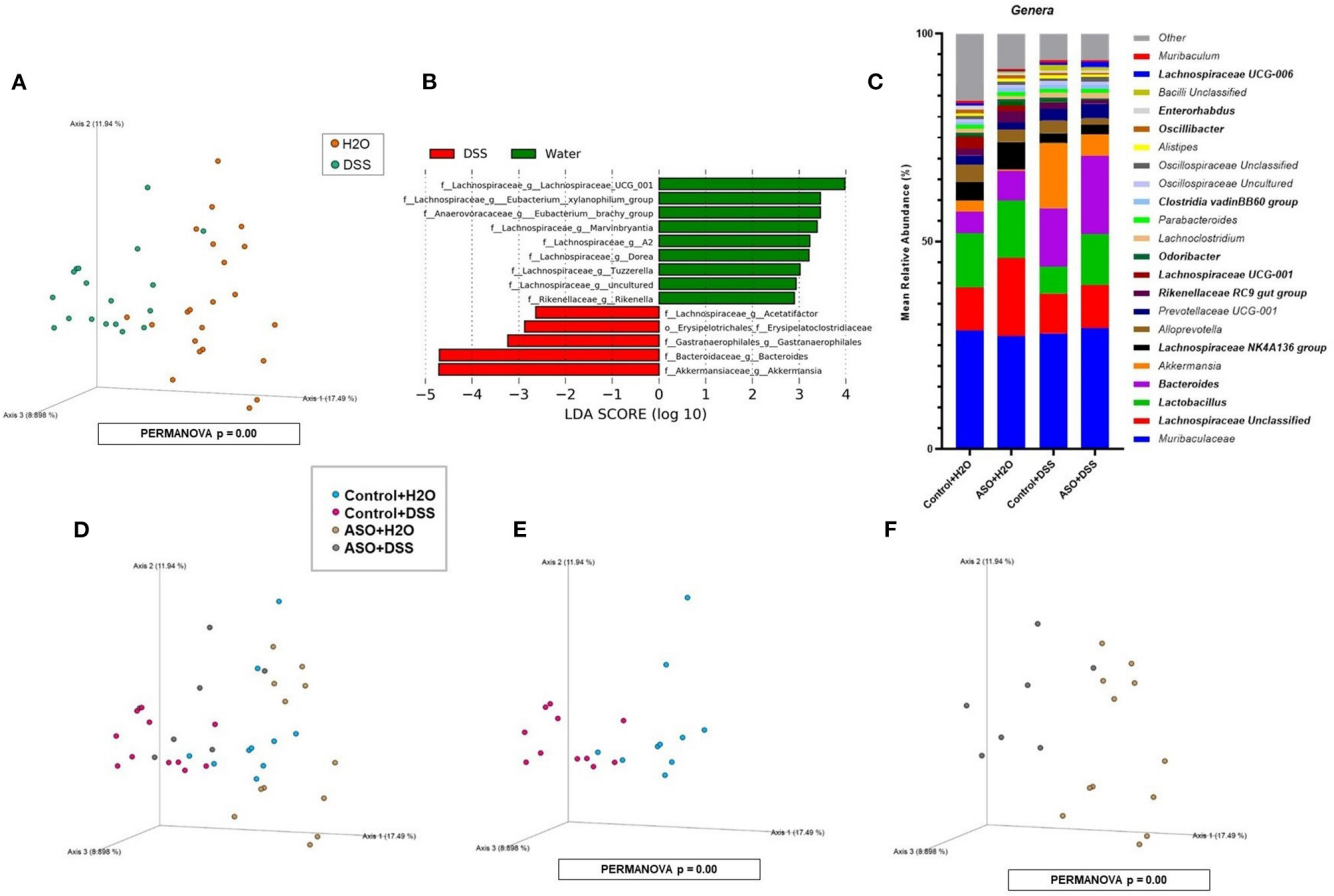
Characterization of fecal microbiota in mice treatment and genotype groups using 16S rRNA sequencing. **(A)** Mice samples clustered separately between treatments, regardless of genotype. **(B)** LEfSe identified bacterial genera clades that are differentially abundant with treatment, irrespective of genotype. Clade colors: H_2_O (green) and DSS (red). Clades in this graph were statistically significant (*p* < 0.05) and exceed an LDA log score of at least (± 2). **(C)** Bar charts of the mean relative abundance of fecal microbial communities at the taxonomic level of genus for mice genotypes with treatments. The mean relative abundance of taxa with >1% average relative abundance are shown. **(D–F)** Fecal microbial communities clustered separately between mice genotypes with treatments. PCoA plots were built on Bray-Curtis dissimilarity metrics. H_2_O, water; DSS, dextran sodium sulfate; ASO, α-synuclein overexpressing.

Microbial communities were examined for an overall treatment effect, regardless of genotype. No significant differences in alpha-diversity indices were observed (MWU: Supplementary Table 1), however between group differences in beta diversity were noted (*q* < 0.00, PCoA: Figure 4A; PERMANOVA: Table 1). Compared to H_2_O-fed mice, DSS-treated mice demonstrated a significant increase in differential abundance for the putative pro-inflammatory genus *Bacteroides*, along with a loss of putative beneficial SCFA-producing genera that included *Lachnospiraceae* (A2; UCG-001; and Uncultured), *Bifidobacterium, Roseburia, Dorea, Marvinbryantia, Eubacterium xylanophilum*, and *Blautia* (*q* < 0.05, Supplementary Tables 2, 3). LEfSe analysis showed that H_2_O-fed mice were associated with multiple putative SCFA-producing bacteria, whereas DSS-treated mice were associated with putative pro-inflammatory bacteria genera *Akkermansia* and *Bacteroides* (Figure 4B). Irrespective of genotype, DSS administration resulted in a robust dysbiotic pro-inflammatory microbial profile characterized by loss of putative SCFA-producing bacteria with a concurrent enrichment in putative pro-inflammatory bacteria.

**Table 1:** Significant differences in intestinal microbial community structures were observed between mice groups in beta diversity analyses conducted on microbial features.

**Mice comparisons**	**Feature taxonomic level**				
	**Sample size**	**Permutations**	**Pseudo-*F***	***p-*value**	***q*-value**
H_2_O vs. DSS	40	9,999	5.53	*0.0001*	**0.0001**
CONTROL+H_2_O vs. CONTROL+DSS	21	9,999	3.28	*0.0001*	**0.0003**
ASO+H_2_O vs. ASO+DSS	19	9,999	3.37	*0.0004*	**0.0008**
CONTROL+H_2_O vs. ASO+H_2_O	22	9,999	1.93	*0.0156*	**0.0187**
CONTROL+DSS vs. ASO+DSS	18	9,999	1.80	*0.0255*	**0.0255**

*PERMANOVA results are based on a Bray-Curtis distance matrix. Significance was determined using 9,999 permutations and corrected for multiple testing using the Benjamini-Hochberg adjusted p-values (q < 0.05 indicated by bold; p < 0.05 indicated by italics)*.

*H_2_O, water; DSS, dextran sodium sulfate; ASO, alpha-synuclein overexpressing*.

*Mice sizes: H_2_O (n = 22); DSS (n = 18); CONTROL+H_2_O (n = 10); CONTROL+DSS (n = 11); ASO+H_2_O (n = 12); ASO+DSS (n = 7)*.

Next, microbial communities were examined for treatment effects within each genotype (i.e., control and ASO). Two-way ANOVA indicated no significant genotype, treatment, or interaction effects for alpha diversity examined at the feature level (data not shown). However, between group differences in beta-diversity in stool microbial community structures were noted (PERMANOVA: Table 1; Figures 4C–F). Microbial communities across all groups were different and dominated by bacteria from the genera *Muribaculaceae, Lachnospiraceae* Unclassified, *Lactobacillus*, and *Bacteroides* (>50% of all sequences; Figure 4C; Supplementary Table 4). DSS administration to control mice significantly increased (*q* < 0.05) the abundance of putative pro-inflammatory genus *Bacteroides* and significantly decreased putative beneficial SCFA-producing genera *Lachnospiraceae* (A2 and UCG-001), *Dorea, Eubacterium xylanophilum*, and *Lactobacillus* (DeSeq2: Table 2). This dysbiotic microbial profile was similarly noted in ASO mice given DSS which increased putative pro-inflammatory genus *Bacteroides*, with a significant decrease (*q* < 0.05) in the abundance of putative beneficial SCFA-producing genera, including *Lachnospiraceae* (A2; UCG-001; Uncultured; and Unclassified), *Dorea, Eubacterium xylanophilum, Marvinbryantia, Anaerotruncus, Dorea*, and *Blautia* (DeSeq2: Table 2). Overall, DSS induced microbiota dysbiosis in both control and ASO mice; however, the ASO mice given DSS showed a greater loss of beneficial SCFA-producing bacteria, than control mice given DSS.

**Table 2:** Genus taxonomic level differential abundance DeSeq2 analysis between control or ASO mice treated with water and dextran sodium sulfate.

**(Phylum) *Genus***	**Base mean**	**Log2 FC**	***p*-value**	***q*-value**
**Control+DSS over Control+H**_**2**_ **O**				
(Firmicutes) *Erysipelatoclostridiaceae* Unclassified	10.56	6.50	*8.78E-05*	**0.002**
(Cyanobacteria) *Gastranaerophilales*	57.99	1.72	*0.009*	0.096
(Bacteroidota) *Bacteroides*	2,400.21	1.34	*0.002*	**0.026**
(Firmicutes) *Clostridia vadinBB60 group*	171.38	0.87	*0.031*	0.229
(Actinobacteriota) *Enterorhabdus*	152.25	−1.12	*0.004*	**0.048**
(Proteobacteria) *Parasutterella*	33.20	−1.80	*0.010*	0.096
(Firmicutes) *[Eubacterium] brachy group*	5.43	−1.88	*0.031*	0.229
(Desulfobacterota) *Desulfovibrio*	88.80	−2.27	*4.52E-04*	**0.007**
(Firmicutes) *Lactobacillus*	4,531.14	−2.77	*2.44E-04*	**0.005**
(Actinobacteriota) *Atopobiaceae* unclassified	5.44	−4.17	*0.017*	0.150
(Firmicutes) *[Eubacterium] xylanophilum group*	68.83	−4.36	*0.003*	**0.041**
(Firmicutes) *Lachnospiraceae* UCG-001	246.12	−8.33	*4.45E-07*	**1.54E-05**
(Firmicutes) *Lachnospiraceae A2*	49.43	−9.70	*3.68E-09*	**1.92E-07**
(Firmicutes) *Dorea*	35.45	−9.95	*1.17E-10*	**1.22E-08**
**ASO+DSS over ASO+H**_**2**_ **O**				
(Firmicutes) *Tyzzerella*	0.54	4.12	*0.047*	0.200
(Firmicutes) *Erysipelotrichaceae*	2.21	3.39	*0.011*	0.073
(Firmicutes) *Lachnospiraceae UCG-006*	109.28	2.55	*9.50E-05*	**1.80E-03**
(Bacteroidota) *Bacteroides*	2,400.21	1.22	*0.010*	0.073
(Firmicutes) *Colidextribacter*	92.93	−0.82	*0.045*	0.204
(Firmicutes) *Lachnospiraceae* Unclassified	2,717.42	−1.02	*0.005*	**0.045**
(Firmicutes) *Oscillibacter*	155.29	−1.03	*0.024*	0.127
(Bacteroidota) *Rikenellaceae RC9 gut group*	442.34	−1.77	*0.002*	**0.026**
(Firmicutes) *Lachnospiraceae NK4A136 group*	930.44	−1.84	*0.029*	0.142
(Firmicutes) *Ruminococcaceae* Unclassified	35.57	−1.87	*0.001*	**0.016**
(Bacteroidota) *Odoribacter*	226.57	−2.11	*0.024*	0.127
(Desulfobacterota) *Bilophila*	21.61	−2.41	*0.015*	0.099
(Campilobacterota) *Helicobacter*	28.16	−2.91	*0.03*	0.142
(Firmicutes) *Blautia*	55.79	−3.84	*3.29E-05*	**7.82E-04**
(Firmicutes) *Dorea*	16.54	−4.07	*0.003*	**0.033**
(Firmicutes) *Anaerotruncus*	6.98	−4.18	*0.003*	**0.033**
(Firmicutes) *Erysipelotrichaceae* Unclassified	4.00	−4.29	*0.022*	0.127
(Firmicutes) *Marvinbryantia*	64.65	−5.38	*0.001*	**0.014**
(Firmicutes) *Lachnospiraceae* Uncultured	13.63	−5.69	*3.65E-06*	**1.73E-04**
(Firmicutes) *Lachnospiraceae UCG-001*	246.12	−6.54	*1.30E-04*	**0.002**
(Firmicutes) *Lachnospiraceae A2*	49.43	−7.36	*5.85E-06*	**1.85E-04**
(Firmicutes) *[Eubacterium] xylanophilum group*	68.83	−8.86	*9.04E-09*	**8.59E-07**

*DeSeq2: Taxa shown have adjusted p-values (q < 0.05 indicated by bold; p < 0.05 indicated by italics). Base Mean is the mean of normalized samples. Log2FC, Log2 fold change of taxa in Control or ASO+DSS mice in comparison to Control or ASO+H_2_O mice samples*.

*H_2_O, water; DSS, dextran sodium sulfate; ASO, alpha-synuclein overexpressing*.

*Control+H_2_O (n = 10); Control+DSS (n = 11); ASO+H_2_O (n = 12); ASO+DSS (n = 7)*.

### Effect of DSS on Motor Function

**Figure 5:**
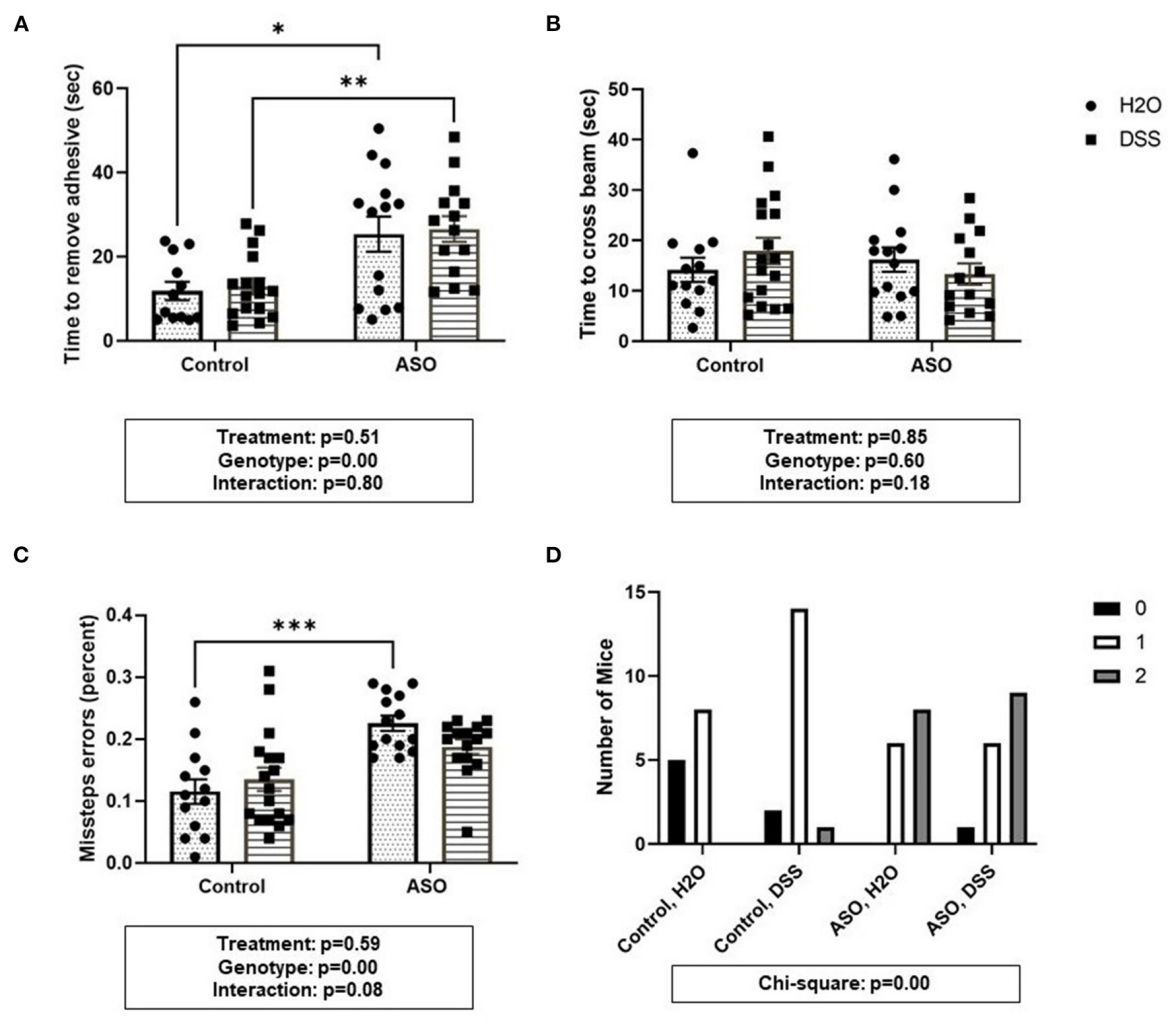
Parkinsonian behavioral analysis in control and ASO mice. Motor function was evaluated using four behaviors. **(A)** Adhesive removal: ASO mice significantly took longer to remove the adhesive from the bridge of the nose than WT mice. **(B)** Beam traverse: There was no difference in the time to cross the beam between WT and ASO mice. **(C)** ASO mice had significantly more missteps off the beam than WT mice. **(D)** Hindlimb Clasp Reflex Score: ASO mice had significantly worse hindlimb clasp than WT littermates. Outliers were omitted prior to analysis (>2 standard deviations from the mean). Two-way ANOVA was conducted and values for different factors are indicated in the graphs followed by *post-hoc* Tukey which is indicated on each graph when appropriate *p < 0.05, **p < 0.01, ***p < 0.001. **(D)** Chi-square analysis. ASO, α-synuclein overexpressing; H_2_O, water; DSS, dextran sodium sulfate.

Time to remove an adhesive from the nasal bridge was significantly impacted by genotype, although DSS-induced intestinal barrier dysfunction did not alter this behavior (Figure 5A: two-way ANOVA: genotype *p* < 0.00, treatment *p* = 0.51, interaction *p* = 0.80). There were no significant differences in time to cross the beam (Figure 5B: two-way ANOVA: genotype *p* = 0.60, treatment *p* = 0.85, interaction *p* = 0.18). However, evaluating the number of errors (i.e., stepping off the beam) revealed a significant effect of genotype wherein ASO mice had significantly greater missteps than control mice, but this was unaltered by DSS-induced intestinal barrier dysfunction (Figure 5C: two-way ANOVA: genotype *p* < 0.00, treatment *p* = 0.59, interaction *p* = 0.08). Finally, Chi-square analysis of the hindlimb clasping score indicated that more ASO mice had impaired motor function compared to control mice (Figure 5D: Chi-square *p* < 0.00). Taken together, a genotype- specific effect was found in three of the behavioral outcomes including adhesive removal, missteps in beam crossing, and hindlimb clasping reflex score. However, DSS administration did not impact motor function.

### Effect of DSS on Brain-Specific PD-Like Outcomes

**Figure 6:**
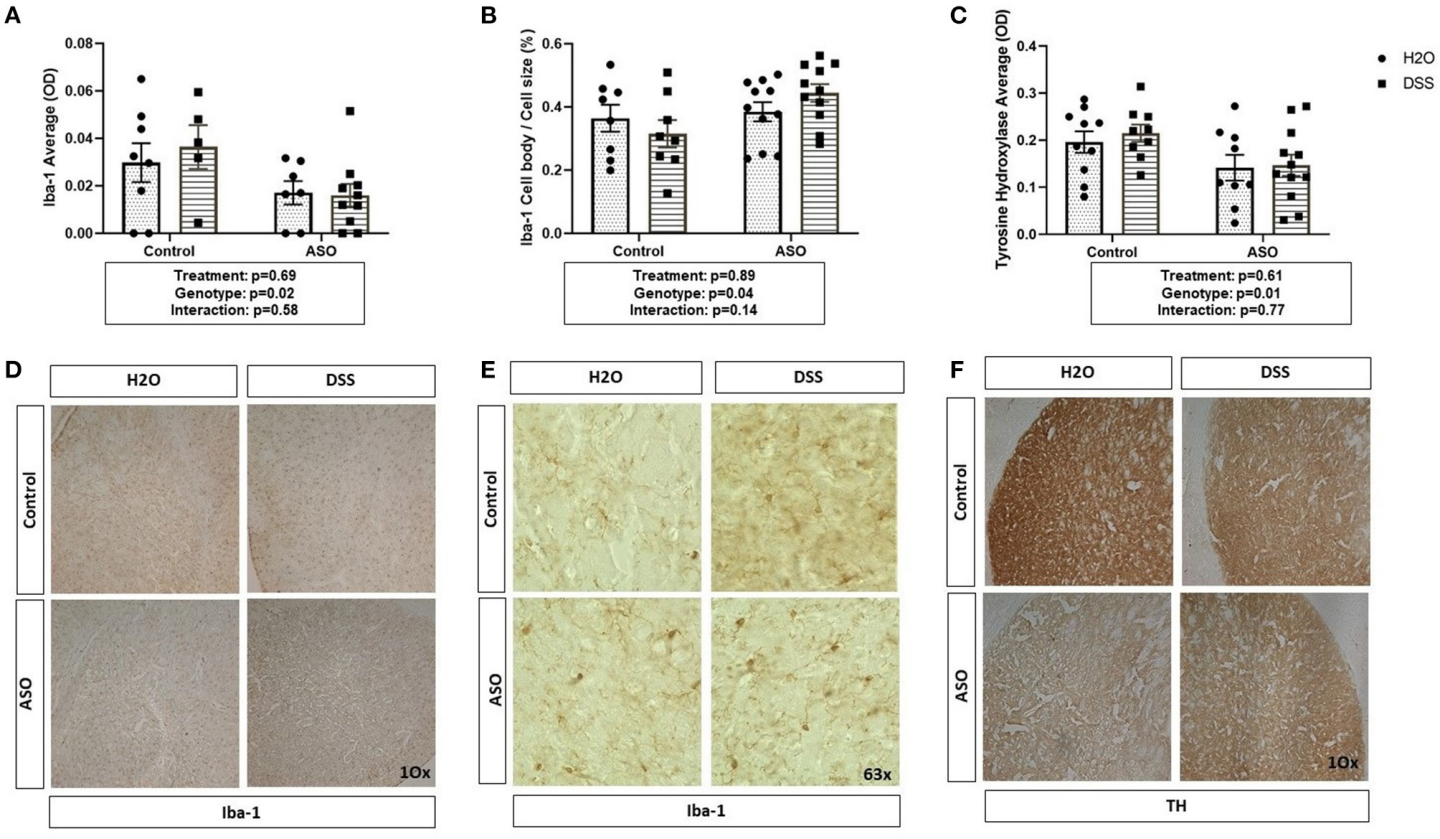
Brain related PD outcome measures exhibit genotypic ASO but not DSS treatment effects. Mice were evaluated for brain pathology. **(A)** ASO mice had a significantly lower amount of microglia than controls. **(B)** ASO mice had significantly more activated microglia than WT. **(C)** ASO mice had a significantly less TH than control. **(D)** Representative images of Iba-1 positive microglia at 10x magnification. **(E)** Representative images of Iba-1 positive microglia at 63x magnification. **(F)** Representative images of TH positive dopaminergic terminals at 10x magnification Outliers were omitted prior to analysis (>2 standard deviations from the mean). **(A–C)** Two-way ANOVA was conducted and values for different factors are indicated in the graphs followed by *post-hoc* Tukey (no *post hoc* significance was identified). ASO, α-synuclein overexpressing; H_2_O, water; DSS, dextran sodium sulfate; TH, Tyrosine hydrolase; Iba-1, Ionized calcium binding adaptor molecule 1.

There was a significant impact of genotype on Iba-1 with levels being lower in ASO mice compared to control mice; however, DSS administration did not impact Iba-1 (Figure 6A: two-way ANOVA: genotype *p* = 0.02, treatment *p* = 0.69, interaction *p* = 0.58). However, perhaps more important than evaluating the presence of microglia (i.e., Iba-1 optical density) is microglia morphology. Non-activated microglia morphologically are ramified in shape. Once microglia are activated (e.g., in response to damaged cells, bacterial products), they retract their processes and take on an ameboid morphology with includes an increase in cell body size (40, 41, 75). Assessing cell body cell size revealed a genotype-specific significant difference between control and ASO mice with greater activated Iba-1 positive microglia in ASO mice; however, there was no impact of DSS administration on microglial morphology (Figure 6B: two-way ANOVA: genotype *p* = 0.04, treatment *p* = 0.89, interaction *p* = 0.14). ASO mice had significantly lower levels of TH staining compared to controls, however DSS treatment did not impact TH staining (Figure 6C: two-way ANOVA: genotype *p* = 0.01, treatment *p* = 0.61, interaction *p* = 0.77). We have included representative images of Iba-1 (Figures 6D,E) and TH (Figure 6F) that was used for analysis. These data demonstrate the ASO mice have fewer microglia than controls, a distinct microglia phenotype compared to controls, and fewer dopaminergic terminals than control mice. However, there is no evidence that these PD-like brain outcomes were impacted by DSS.

### Effect of DSS on Bacterial Translocation and Systemic Inflammation

**Figure 7:**
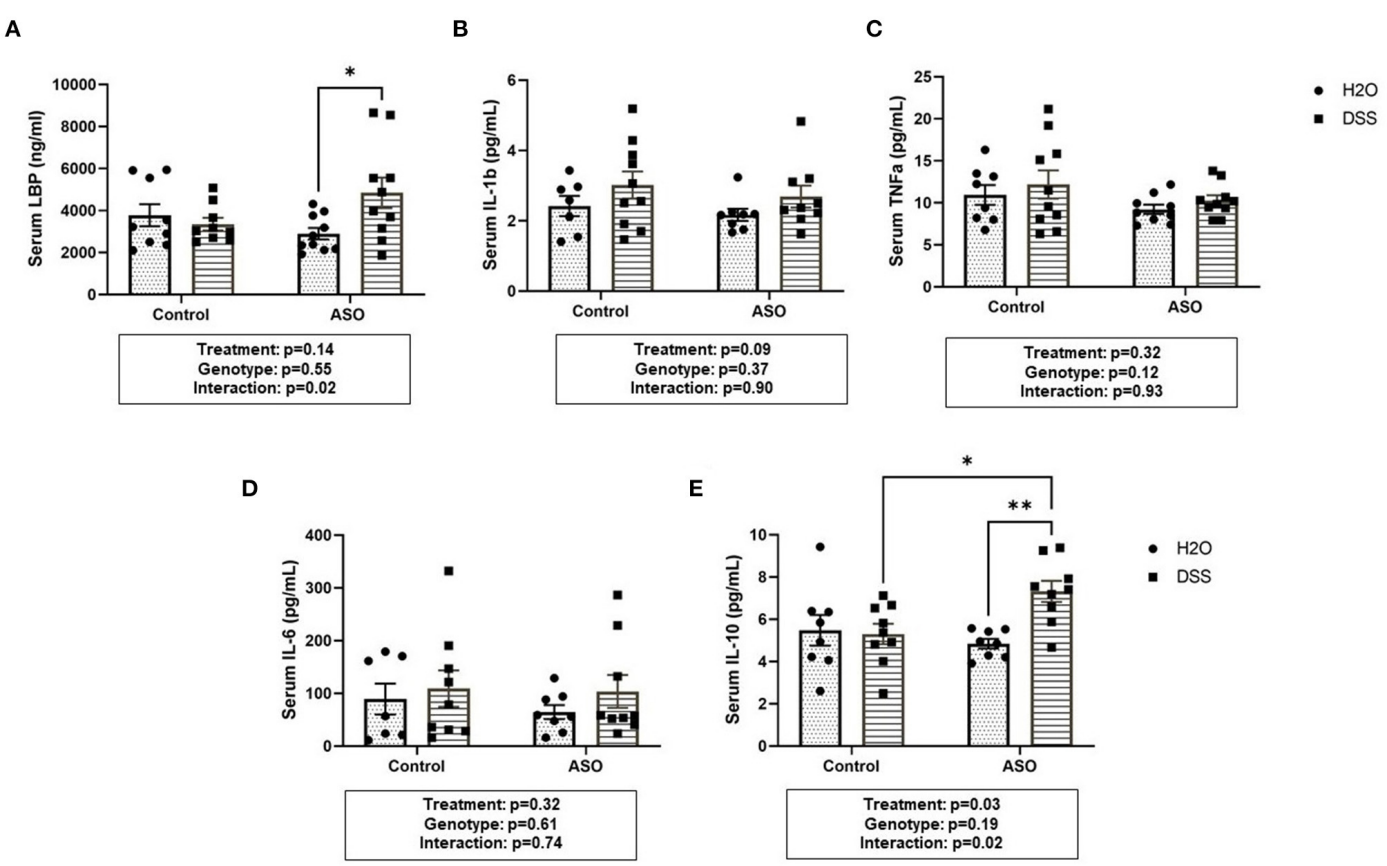
Systemic Inflammation Markers. **(A)** Serum LBP was higher in ASO mice that received DSS although this failed to reach statistical significance. However, there was significance in the interaction between genotype and treatment (*p* = 0.0240). Data are presented as means (ng/ml). There was no significant elevation of systemic inflammatory cytokines in DSS treated control or ASO mice including **(B)** IL-1b, **(C)** TNFa, and **(D)** IL-6 the three key markers in systemic inflammation. **(E)** Only DSS treated ASO mice displayed significantly (*p* = 0.0328) increased serum IL-10. Outliers were omitted prior to analysis (>2 standard deviations from the mean). Two-way ANOVA was conducted and values for different factors are indicated in the graphs followed by *post-hoc* Tukey which is indicated on each graph when appropriate *p < 0.05, **p < 0.01. ASO, α-synuclein overexpressing; LBP, Lipopolysaccharide binding protein.

DSS administration significantly increased serum LBP in ASO mice, an effect that was not observed in control mice (Figure 7A: two-way ANOVA: genotype *p* = 0.55, treatment *p* = 0.14, interaction *p* = 0.02). This suggests that the host immune response to barrier dysfunction is different in control and ASO mice. Despite the increase in LBP, none of the pro-inflammatory cytokines evaluated in the serum were increased by DSS administration (nor were they impacted by genotype) including IL-1β, TNF-α, or IL-6 (Figures 7B–D). Paradoxically, DSS-induced intestinal barrier dysfunction increased serum IL-10 in ASO mice (Figure 7E: two-way ANOVA: genotype *p* = 0.19, treatment *p* = 0.03, interaction *p* = 0.02). IL-10 is generally considered an anti-inflammatory cytokine, and this may (at least partially) represent a compensatory mechanism that may have prevented DSS-induced barrier dysfunction from promoting PD-like behavior and brain pathology. Taken together, these data indicate that ASO mice given DSS have higher levels of LBP than control mice and ASO mice given water which may reflect the increase in pro-inflammatory LPS-containing bacteria in this group. Despite this increase there was not an increase in pro-inflammatory cytokines, but the anti-inflammatory cytokine IL-10 was increased.

### Relationship Between Intestinal Outcomes and Motor Function / Brain Pathology

Despite being inbred and genetically similar, outcomes reflect heterogenous outcomes in terms of intestinal barrier dysfunction, intestinal and systemic inflammation. Thus, it is conceivable that those mice with the highest DSS-induced intestinal barrier dysfunction, intestinal inflammation, microbiota dysbiosis, and systemic inflammation may correspondingly show greater motor dysfunction and brain pathology. However, none of the intestinal outcomes nor systemic inflammation significantly correlated with motor function or brain outcomes (Supplementary Table 5).

## Discussion

There are numerous different pathways by which the gut could contribute to PD and in this study, we evaluated the contribution of intestinal hyperpermeability to the PD-like phenotype based on the following rationale: (1) data from our group has demonstrated intestinal hyperpermeability is observed in PD patients (8, 76, 77), (2) numerous conditions that may be risk factors for PD (e.g., diabetes, ulcerative colitis) are associated with intestinal hyperpermeability (78, 79), and (3) intestinal hyperpermeability is associated with increased systemic inflammation which may drive neuroinflammation in PD (80, 81). Thus, the rationale behind this study was to investigate if increased intestinal permeability could promote neuroinflammation and exacerbate the PD phenotype in ASO mice through a mechanism including intestinal and/or systemic inflammation.

Administration of DSS in drinking water is a well-established rodent model to induce intestinal barrier dysfunction, intestinal inflammation, and pro-inflammatory changes in the intestinal microbiota (82, 83). This study demonstrated that the low dose (1%) DSS was sufficient to cause intestinal (colonic) barrier dysfunction and intestinal microbiota dysbiosis, but only mild intestinal inflammation without systemic inflammation and that this was not sufficient to worsen PD-like brain pathology nor motor function in ASO mice.

This finding appears to be in contradiction to prior studies demonstrating that a high dose of DSS causes marked intestinal inflammation and exacerbates the PD-like phenotype in rodent toxin models of PD. One recent study combined administration of 2.5% DSS treatment with paraquat/LPS and found that DSS exacerbates LPS/paraquat effects on microglial activation (84). Houser et al. used 2% DSS and demonstrated showed worsening of PD-like brain pathology induced by MPTP (85). Higher doses of DSS (2–2.5%) are well-established to cause severe intestinal and systemic inflammation. The difference between these prior studies and this current report suggests that overt intestinal inflammation (high levels of stool calprotectin) and/or systemic inflammation may be required to promote the PD-like phenotype in ASO mice, but such a conclusion will require additional investigation (e.g., DSS dose response). However, differences in microbiota communities between institutions [so “cage effects” (86)] may also partially account for differences between studies as the microbiota can dictate the response to intestinal disruptors such as alcohol, stress, and non-steroidal anti-inflammatory medications (87).

The concept that intestinal inflammation is a key feature in promoting PD is supported by observations in humans. Epidemiological studies demonstrate that inflammatory bowel disease (IBD) is a risk factor for PD (29, 79, 88, 89). IBD is characterized by chronic intestinal inflammation, pro-inflammatory dysbiosis and intestinal leak (27, 28, 90). Treatment of IBD patients with biologics (e.g., TNF antibody) that effectively control intestinal inflammation and induce remission in IBD patients, reduces risk of PD (despite most patients still having intestinal barrier dysfunction and microbiota dysbiosis) (91). This evidence supports that intestinal inflammation is a critical feature mediating the effects of the intestinal barrier and intestinal microbiota on PD.

A few findings observed in ASO mice require additional discussion. First, despite having similar levels of intestinal barrier dysfunction ASO mice had higher levels of LBP. This can be explained in one of two ways: (1) DSS treatment had different impact on microbiota function in ASO mice compared to control mice leading to release of more LPS in ASO mice or other metabolic impact that is not reflect in microbiota composition (92, 93) or (2) differences in LBP levels could reflect differences in hepatic immune response to intestinal barrier dysfunction. Second, the finding that serum IL-10 was increased in only DSS-treated ASO mice was unexpected because DSS caused intestinal barrier dysfunction in both ASO and control mice. But elevated serum IL-10 along with elevated serum LBP in DSS-treated ASO mice suggest that immune response to the inflammatory trigger in ASO mice is different than control mice. This possibility is supported by recent studies in patients with PD who have dysregulated and exaggerated immune/inflammatory signaling pathways (94). Future studies are required to directly test this hypothesis in ASO mice.

There are some study limitations worth noting. There is no ideal animal model for PD and each model recapitulates only some aspects of PD. In this study, a genetic model of misfolded α-synuclein was used but the effects of low dose DSS should be studied in other PD models such as transgenic mice that overexpress human α-synuclein with a PD-associated mutation (A53T) (95), *Parkin* knockout mice (96, 97), and the mitopark mouse model (98, 99) could all be considered for future studies. Additionally, this study administered DSS in three cycles to mimic chronic, intermittent barrier dysfunction; future studies could either use a higher dose of DSS (e.g., 2% DSS) or extend this treatment period to four to five cycles to determine if longer duration would be sufficient to trigger more severe intestinal barrier dysfunction / inflammation and promote the PD-like phenotype. Another consideration is how within group variability and between institution differences in microbiota may be modifying the response to low dose DSS. Although these mice are genetically identical there clearly is variability in the response to DSS. The low dose of DSS likely contributed to the variability (as opposed to a higher dose that would induce robust barrier dysfunction associated with severe intestinal inflammation). However, this variability also represents what happens in the population insomuch as individuals have a different response to the same “disruptor” examples include alcohol (100–102), stress (103, 104), NSAID (105, 106) so the variability could be viewed as a strength in that it models individual susceptibility.

To the best of our knowledge, no studies have investigated the role of intestinal permeability without severe intestinal inflammation in rodent models of PD to determine whether intestinal inflammation is a critical element of the gut-brain axis in the PD pathogenesis. This study provides a significant step forward in our understanding of the role of intestinal permeability, intestinal inflammation and the gut microbiome in the gut-brain axis and PD insomuch as intestinal and systemic inflammation appear to be key features mediating the impact of the intestine on the brain in (at least) the ASO PD model.

## Data Availability Statement

The raw sequence data supporting the findings have been deposited in the NCBI Sequence Read Archive under BioProject PRJNA781983. Further queries should be directed to the corresponding author(s).

## Ethics Statement

The animal study was reviewed and approved by Rush University Institutional Animal Care and Use Committee.

## Author Contributions

AK, CF, RV, and AJ: conceptualization and study design. AJ, MS, RV, PE, AN, and SG: data analysis. AJ, PE, MS, SW, DF, SR, BB, and AP: data collection. AK and RV: resources. AJ, AK, CF, RV, SG, PE, and AN: writing-original draft. AK, CF, and RV: supervision. All authors: writing-review and editing. All authors contributed to the article and approved the submitted version.

## Funding

AK would like to acknowledge philanthropy funding from Barbara and Larry Field, Ellen and Philip Glass, and Marcia and Silas Keehn as well as funding from the Department of Defense (W81XWH-17-1-0587 to AK), the National Institutes of Health including National Institute on Alcohol Abuse and Alcoholism (R24AA026801 to AK), and the National Institute of Aging (R01AG056653 to RV).

## Conflict of Interest

The authors declare that the research was conducted in the absence of any commercial or financial relationships that could be construed as a potential conflict of interest.

## Publisher's Note

All claims expressed in this article are solely those of the authors and do not necessarily represent those of their affiliated organizations, or those of the publisher, the editors and the reviewers. Any product that may be evaluated in this article, or claim that may be made by its manufacturer, is not guaranteed or endorsed by the publisher.
