# Supplementary material for: Intestinal Barrier Dysfunction in the Absence of Systemic Inflammation Fails to Exacerbate Motor Dysfunction and Brain Pathology in a Mouse Model of Parkinson's Disease
Source: Front Neurol. 2022 May 18;13:882628. doi: 10.3389/fneur.2022.882628 (PMC9159909; doi:10.3389/fneur.2022.882628)
Supplement: Supplementary file 1 [file Table_1.DOCX]

Supplementary Material

| **Supplementary Table 1.** Alpha-diversity values of mice treatments, regardless of genotype. | | | | |
| --- | --- | --- | --- | --- |
| **Mice Comparisons** | **Diversity Index** | **H2O**  **Mean ± (SD)** | **DSS**  **Mean ± (SD)** | ***p*-value** |
| H2O vs DSS | Shannon | 5.66 (0.90) | 5.49 (0.35) | 0.240 |
| H2O vs DSS | Simpson | 0.93 (0.07) | 0.94 (0.02) | 0.367 |
| H2O vs DSS | Observed Features | 189.86 (45.50) | 181.28 (24.22) | 0.480 |
| H2O vs DSS | Evenness | 0.75 (0.10) | 0.73 (0.04) | 0.132 |
| Mann-Whitney U test: Alpha diversity values of Shannon’s Index, Simpson’s Index, Observed Features and Pielou’s Evenness were examined at the feature level. Rarefaction level at 19,000 sequences per sample. Mean index score and standard deviation (SD) are displayed. H2O = water; DSS = dextran sodium sulfate. Mice sizes: H2O (n=22); DSS (n=18). | | | | |

| **Supplementary Table 2.** Relative abundances of bacterial taxa altered between mice treatments, regardless of genotype. | | |
| --- | --- | --- |
| **Taxonomic Level** | **H2O: Mean RA % ± (SD) %** | **DSS: Mean RA % ± (SD) %** |
| **Phylum** | | |
| Bacteroidota | 45.44 **±** (13.3) | 54.05 ± (11.22) |
| Firmicutes | 49.74 **±** (12.82) | 32.12 ± (7.79) |
| Verrucomicrobiota | 1.5 **±** (4.13) | 11.64 ± (9.22) |
| Actinobacteriota | 1.13 **±** (1.08) | 0.57 ± (0.26) |
| Proteobacteria | 0.98 **±** (2.95) | 0.56 ± (0.69) |
| Desulfobacterota | 0.59 **±** (0.47) | 0.33 ± (0.49) |
| ***Genus*** | | |
| *Muribaculaceae* | 27.83 **±** (8.86) | 28.37 **±** (8.38) |
| *Lachnospiraceae* Unclassified | 14.99 **±** (10.93) | 9.87 **±** (4.37) |
| *Lactobacillus* | 13.48 **±** (16.66) | 8.77 **±** (7.04) |
| *Bacteroides* | 6.17 **±** (3.53) | 15.90 **±** (9.08) |
| *Akkermansia* | 1.50 **±** (4.13) | 11.64 **±** (9.22) |
| *Lachnospiraceae NK4A16 group* | 5.58 **±** (4.10) | 2.29 **±** (1.94) |
| *Alloprevotella* | 3.55 **±** (5.44) | 2.51 **±** (4.26) |
| *Prevotellaceae UCG-001* | 1.97 **±** (1.20) | 3.07 **±** (1.58) |
| *Rikenellaceae RC9 gut group* | 2.21 **±** (1.65) | 1.32 **±** (1.00) |
| *Lachnospiraceae UCG-001* | 2.00 **±** (2.83) | 0.00 **±** (0.01) |
| *Odoribacter* | 1.31 **±** (1.27) | 0.77 **±** (1.09) |
| *Lachnoclostridium* | 0.84 **±** (0.51) | 1.32 **±** (0.99) |
| *Parabacteroides* | 1.02 **±** (0.77) | 0.83 **±** (0.67) |
| *Clostridia vadinBB60 group* | 0.71 **±** (0.67) | 1.03 **±** (0.71) |
| *Oscillospiraceae* Uncultured | 0.78 **±** (0.77) | 0.82 **±** (0.51) |
| *Oscillospiraceae* Unclassified | 0.77 **±** (0.56) | 0.81 **±** (0.76) |
| *Alistipes* | 0.69 **±** (0.48) | 0.79 **±** (0.57) |
| *Oscillibacter* | 0.89 **±** (0.61) | 0.52 **±** (0.32) |
| *Enterorhabdus* | 0.69 **±** (0.32) | 0.55 **±** (0.25) |
| *Bacilli* Unclassified | 0.19 **±** (0.48) | 1.08 **±** (1.81) |
| *Lachnospiraceae UCG-006* | 0.30 **±** (0.51) | 0.93 **±** (1.24) |
| *Muribaculum* | 0.55 **±** (0.42) | 0.46 **±** (0.41) |
| Mean RA % = average number of sequences per taxa, calculated from the total sum of all sequence counts, depicted as a percentage. (SD) % = standard deviation as a percentage. Microbial taxa (˃ 1%) shown. H2O = water; DSS = dextran sodium sulfate. Mice sizes: H2O (n=22); DSS (n=18). | | |

| **Supplementary Table 3.** Genus taxonomic level differential abundance DeSeq2 analysis between mice treatments, regardless of genotype. | | | | |
| --- | --- | --- | --- | --- |
| **(Phylum) *Genus*** | **Base Mean** | **Log2FC**  **(DSS/H2O)** | ***p*-value** | ***q*-value** |
| (Firmicutes) *Erysipelatoclostridiaceae* Unclassified | 12.42 | 6.21 | *2.42E-10* | **3.97E-09** |
| (Firmicutes) *Turicibacter* | 9.90 | 6.17 | *0.003* | **0.016** |
| (Firmicutes) *[Eubacterium] siraeum group* | 1.63 | 4.61 | *0.030* | **0.088** |
| (Cyanobacteria) *Gastranaerophilales* | 57.99 | 1.49 | *0.001* | **0.008** |
| (Firmicutes) *Lachnospiraceae UCG-006* | 109.28 | 1.41 | *0.002* | **0.013** |
| (Proteobacteria) *Rhodospirillales* Uncultured | 62.61 | 1.25 | *0.038* | 0.101 |
| (Bacteroidota) *Bacteroides* | 2400.21 | 1.18 | *6.93E-05* | **7.64E-04** |
| (Firmicutes) *Colidextribacter* | 92.93 | -0.59 | *0.031* | 0.088 |
| (Firmicutes) *Lachnospiraceae* Unclassified | 2717.42 | -0.63 | *0.017* | 0.059 |
| (Bacteroidota) *Rikenellaceae RC9 gut group* | 442.34 | -0.86 | *0.036* | 0.099 |
| (Firmicutes) *Oscillibacter* | 155.29 | -0.88 | *0.003* | **0.013** |
| (Desulfobacterota) *Desulfovibrio* | 88.80 | -1.27 | *0.010* | **0.041** |
| (Firmicutes) *Ruminococcaceae* Unclassified | 35.57 | -1.34 | *0.001* | **0.005** |
| (Firmicutes) *Lachnospiraceae NK4A136 group* | 930.44 | -1.35 | *0.019* | 0.063 |
| (Firmicutes) *[Eubacterium] brachy group* | 5.43 | -1.49 | *0.018* | 0.060 |
| (Firmicutes) *Clostridia* Unclassified | 15.07 | -1.51 | *0.020* | 0.063 |
| (Firmicutes) *Blautia* | 55.79 | -1.77 | *0.007* | **0.030** |
| (Desulfobacterota) *Bilophila* | 21.61 | -1.78 | *0.010* | **0.040** |
| (Firmicutes) *Tuzzerella* | 5.57 | -1.96 | *0.041* | 0.106 |
| (Bacteroidota) *Rikenella* | 21.94 | -2.33 | *0.027* | 0.081 |
| (Firmicutes) *Anaerotruncus* | 6.98 | -2.55 | *0.016* | 0.059 |
| (Firmicutes) *Lachnospiraceae* Uncultured | 13.63 | -3.31 | *2.62E-05* | **3.57E-04** |
| (Actinobacteriota) *Atopobiaceae* Unclassified | 5.44 | -4.13 | *0.003* | **0.013** |
| (Proteobacteria) *Acinetobacter* | 1.91 | -4.29 | *0.045* | 0.113 |
| (Firmicutes) *Marvinbryantia* | 64.65 | -4.54 | *1.32E-04* | **0.001** |
| (Firmicutes) *Erysipelotrichaceae* Unclassified | 2.83 | -4.77 | *0.001* | **0.005** |
| (Firmicutes) *Dorea* | 16.54 | -5.70 | *7.06E-13* | **1.93E-11** |
| (Firmicutes) *Roseburia* | 21.49 | -6.07 | *7.45E-05* | **7.64E-04** |
| (Actinobacteriota) *DNF00809* | 3.75 | -6.13 | *0.001* | **0.008** |
| (Firmicutes) *[Eubacterium] xylanophilum group* | 68.83 | -6.14 | *1.10E-10* | **2.25E-09** |
| (Actinobacteriota) *Bifidobacterium* | 29.20 | -6.49 | *6.93E-04* | **0.005** |
| (Firmicutes) *Lachnospiraceae UCG-001* | 246.12 | -9.58 | *1.510E-18* | **6.189E-17** |
| (Firmicutes) *Lachnospiraceae A2* | 49.43 | -10.59 | *9.480E-19* | **6.189E-17** |
| DeSeq2: Taxa shown have adjusted *p*-values (*q* < 0.05 indicated by bold; *p* < 0.05 indicated by italics). Base Mean is the mean of normalized samples. Log2FC = Log2 fold change of taxa in DSS-treated mice in comparison to H2O-treated mice fecal samples. H2O-treated (n=22); DSS-treated (n=18) mice groups. | | | | |

| **Supplementary Table 4.** Relative abundances of bacterial taxa altered among mice groups. | | | | |
| --- | --- | --- | --- | --- |
| **Taxonomic Level** | **Control + H2O:**  **Mean RA% ± (SD)%** | **ASO + H2O:**  **Mean RA% ± (SD)%** | **Control + DSS:**  **Mean RA% ± (SD)%** | **ASO + DSS:**  **Mean RA% ± (SD)%** |
| **Phylum** | | | | |
| Bacteroidota | 45.28 **±** (17.26) | 45.57 ± (9.68) | 52.63 ± (11.59) | 56.28 ± (11.08) |
| Firmicutes | 47.54 **±** (16.66) | 51.57 ± (8.87) | 29.45 ± (6.25) | 36.32 ± (8.56) |
| Verrucomicrobiota | 2.72 **±** (5.79) | 0.48 ± (1.65) | 15.72 ± (7.66) | 5.24 ± (8.03) |
| Actinobacteriota | 1.74 **±** (1.39) | 0.62 ± (0.21) | 0.54 ± (0.24) | 0.62 ± (0.30) |
| Proteobacteria | 1.94 **±** (4.28) | 0.18 ± (0.10) | 0.72 ± (0.83) | 0.32 ± (0.28) |
| Desulfobacterota | 0.38 **±** (0.23) | 0.77 ± (0.56) | 0.20 ± (0.11) | 0.77 ± (0.52) |
| ***Genus*** | | | | |
| *Muribaculaceae* | 28.55 **±** (10.35) | 27.23 **±** (7.83) | 27.86 **±** (9.80) | 29.16 **±** (6.15) |
| *Lachnospiraceae* Unclassified | 10.45 **±** (6.27) | 18.78 **±** (12.71) | 9.62 **±** (2.65) | 10.26 **±** (6.49) |
| *Lactobacillus* | 13.04 **±** (21.90) | 13.85 **±** (11.70) | 6.46 **±** (4.10) | 12.40 **±** (9.35) |
| *Bacteroides* | 5.13 **±** (3.81) | 7.04 **±** (3.18) | 14.08 **±** (7.52) | 18.77 **±** (11.13) |
| *Akkermansia* | 2.72 **±** (5.79) | 0.48 **±** (1.65) | 15.72 **±** (7.66) | 5.24 **±** (8.03) |
| *Lachnospiraceae NK4A16 group* | 4.39 **±** (3.42) | 6.57 **±** (4.50) | 2.33 **±** (1.38) | 2.22 **±** (2.74) |
| *Alloprevotella* | 4.19 **±** (6.25) | 3.01 **±** (4.88) | 3.07 **±** (4.84) | 1.64 **±** (3.30) |
| *Prevotellaceae UCG-001* | 2.21 **±** (1.40) | 1.77 **±** (1.02) | 2.83 **±** (0.72) | 3.46 **±** (2.44) |
| *Rikenellaceae RC9 gut group* | 1.64 **±** (1.41) | 2.68 **±** (1.74) | 1.59 **±** (1.20) | 0.90 **±** (0.32) |
| *Lachnospiraceae UCG-001* | 2.92 **±** (3.34) | 1.24 **±** (2.18) | 0.00 **±** (0.01) | 0.00 **±** (0.00) |
| *Odoribacter* | 0.99 **±** (1.28) | 1.58 **±** (1.24) | 0.98 **±** (1.24) | 0.43 **±** (0.75) |
| *Lachnoclostridium* | 0.89 **±** (0.58) | 0.80 **±** (0.47) | 1.30 **±** (1.02) | 1.34 **±** (1.01) |
| *Parabacteroides* | 1.06 **±** (0.85) | 0.97 **±** (0.74) | 0.81 **±** (0.62) | 0.86 **±** (0.79) |
| *Clostridia vadinBB60 group* | 0.54 **±** (0.43) | 0.86 **±** (0.81) | 1.09 **±** (0.80) | 0.93 **±** (0.58) |
| *Oscillospiraceae* Uncultured | 0.70 **±** (0.78) | 0.85 **±** (0.79) | 0.81 **±** (0.43) | 0.83 **±** (0.65) |
| *Oscillospiraceae* Unclassified | 0.72 **±** (0.56) | 0.82 **±** (0.59) | 0.64 **±** (0.30) | 1.08 **±** (1.16) |
| *Alistipes* | 0.72 **±** (0.48) | 0.67 **±** (0.50) | 0.90 **±** (0.55) | 0.60 **±** (0.59) |
| *Oscillibacter* | 0.92 **±** (0.63) | 0.86 **±** (0.61) | 0.54 **±** (0.32) | 0.50 **±** (0.35) |
| *Enterorhabdus* | 0.86 **±** (0.36) | 0.55 **±** (0.19) | 0.51 **±** (0.21) | 0.60 **±** (0.31) |
| *Bacilli* Unclassified | 0.11 **±** (0.18) | 0.27 **±** (0.63) | 1.30 **±** (2.08) | 0.72 **±** (1.38) |
| *Lachnospiraceae UCG-006* | 0.47 **±** (0.73) | 0.17 **±** (0.12) | 0.69 **±** (1.09) | 1.31 **±** (1.50) |
| *Muribaculum* | 0.65 **±** (0.51) | 0.47 **±** (0.32) | 0.48 **±** (0.47) | 0.43 **±** (0.35) |
| Mean RA % = average number of sequences per taxa, calculated from the total sum of all sequence counts, depicted as a percentage. (SD) % = standard deviation as a percentage. Microbial taxa (˃ 1%) shown. H2O = water; DSS = dextran sodium sulfate; ASO = *alpha*-synuclein overexpressing. Mice sizes: Control+H2O (n=10), ASO+H2O (n=12), Control+DSS (n=11), and ASO+DSS (n=7). | | | | |

| **Supplementary Table 5.** Pearson correlation analysis between intestinal permeability and brain readouts. | | |
| --- | --- | --- |
| **Brain-Related Outcomes** | ***p*-value** | **Correlation Coefficient (r)** |
| 1. **INTESTINAL PERMEABILITY (S/L RATIO) CORRELATED WITH BRAIN OUTCOMES** | | |
| Beam Transversal | 0.9772 | -0.00 |
| Percent of Missteps | 0.1717 | 0.19 |
| Adhesive Removal | 0.3410 | 0.14 |
| Iba-1 (OD) | 0.6568 | 0.14 |
| Iba-1 (Morphology) | 0.7552 | 0.06 |
| TH (OD) | 0.6386 | 0.08 |
| 1. **OVERT INTESTINAL PERMEABILITY (S/L RATIO) CORRELATED WITH BRAIN OUTCOMES** | | |
| Beam Transversal | 0.1761 | 0.71 |
| Percent of Missteps | 0.1485 | 0.75 |
| Adhesive Removal | 0.0613 | 0.86 |
| Iba-1 (OD) | 0.8847 | 0.18 |
| Iba-1 (Morphology) | 0.2326 | 0.77 |
| TH (OD) | 0.2862 | 0.71 |
| Pearson correlation analysis between (1) overall intestinal permeability and brain outcomes and (2) a subset of DSS-treated ASO mice with the highest intestinal leak and brain readouts. S/L Ratio = sucralose/lactulose ratio; TH = Tyrosine hydrolase; Iba-1 = Ionized calcium binding adaptor molecule | | |
